# Intra-Clutch Ratio of Yolk Progesterone Level Changes with Laying Date in Rockhopper Penguins: A Strategy to Influence Brood Reduction?

**DOI:** 10.1371/journal.pone.0027765

**Published:** 2011-11-16

**Authors:** Maud Poisbleau, Laurent Demongin, Charline Parenteau, Marcel Eens

**Affiliations:** 1 Department of Biology - Ethology, University of Antwerp, Wilrijk, Belgium; 2 Centre d'Etudes Biologiques de Chizé, Centre National de la Recherche Scientifique, Villiers en Bois, France; University of Lethbridge, Canada

## Abstract

Hatching asynchrony in avian species generally leads to a size hierarchy among siblings, favouring the first-hatched chicks. Maternally deposited hormones affect the embryo and chick's physiology and behaviour. It has been observed that progesterone, a hormone present at higher levels than other steroid hormones in egg yolks, is negatively related to body mass in embryos, chicks and adults. A differential within-clutch progesterone deposition could therefore be linked to the size hierarchy between siblings and to the resulting brood reduction. We tested whether yolk progesterone levels differed between eggs according to future parental ability to feed the entire clutch in wild rockhopper penguins *Eudyptes chrysocome*. This species presents a unique reversed egg-size dimorphism and hatching asynchrony, with the larger second-laid egg (B-egg) hatching before the smaller first-laid egg (A-egg). Yolk progesterone levels increased only slightly with female body mass at laying. However, intra-clutch ratios were not related to female body mass. On the other hand, yolk progesterone levels increased significantly with the date of laying onset for A-eggs while they decreased for B-eggs. Early clutches therefore had proportionally more progesterone in the B-egg compared to the A-egg while late clutches had proportionally less progesterone in the B-egg. We propose that females could strategically regulate yolk progesterone deposition within clutches according to the expected food availability during chick growth, an adaptive strategy to adjust brood reduction to conditions. We also discuss these results, relating to yolk progesterone, in the broader context of other yolk steroids.

## Introduction

An individual's experiences of the environment often lead to variation in its growth, condition and physiological state [1]–[3]. Similarly, environmental conditions that organisms experience during early life influence their development and can also have permanent effects in adulthood [4]–[7]. Consequently, maternal state can affect offspring via cytoplasmic factors (e.g. nutrients, hormones and carotenoids) that may influence offspring development [8], [9]. Therefore, prenatal maternal effects may provide a powerful mechanism for adaptive phenotypic plasticity, in which the environment experienced by the mother is translated into phenotypic variation in the offspring [8], [9].

In altricial and semi-altricial birds, asynchronous hatching generally leads to a size hierarchy among siblings, and to a competitive disadvantage for the youngest siblings compared to older ones [10]–[12]. This size hierarchy is thought to optimize the fitness of the parents by facilitating brood reduction when resource availability does not enable parents to feed the entire clutch properly [13]–[15]. In this context, females should adjust prenatal maternal effects over the laying sequence, and thus increase or offset the chick's disadvantage in hatching later, according to breeding conditions such as the parents' future ability to feed the chicks. Females unable to bear the cost of feeding a large clutch should give an advantage to the first egg to hatch in order to accelerate brood reduction. For instance, avian mothers deposit hormones differentially among their eggs, and these hormones have been shown consequently to affect the embryo and chick's physiology and behaviour (see reviews in Groothuis & von Engelhardt [16], Gil [17] and Groothuis & Schwabl [18]). It has therefore been proposed that elevated yolk hormone levels may modulate sibling competition and facilitate brood reduction under unfavourable conditions in birds (see review in [19]). Several studies have suggested that intra-clutch variation in yolk testosterone and yolk corticosterone can act as an adaptive maternal effect in altricial species where modulation of competition between siblings would benefit mothers (see for example [20], [21]). However, in precocial species where mothers would not benefit from a modulation of sibling quality, intra-clutch variation in yolk hormones may play little or no adaptive role (see for example [20], [22]).

According to Lipar et al. [23], the patterns of hormone deposition (progesterone, testosterone and estradiol), in terms of timing and quantity, in yolk of altricial species (dark-eyed junco *Junco hyemalis* and red-winged blackbird *Agelaius phoeniceus*) matched those predicted by previous studies of the temporal patterns of steroidogenesis in the follicle of the domestic hens *Gallus gallus*, a precocial species [24]. We can therefore assume that the pattern of yolk hormone deposition is relatively similar between altricial and precocial species. Variations in yolk steroid levels among and within clutches are then not simply by-products of the steroid levels in the plasma of the female. Moreover, as steroid levels can vary independently between the follicle and the plasma, it has been suggested that the follicle is the primary influence on the pattern of steroid deposition within an individual yolk (see [23]).

Progesterone is mainly produced during yolk formation by granulosa cells of the preovulary follicle [25]. This hormone is present in yolks of freshly laid eggs at higher levels than the other steroids [23], [26], [27]. Progesterone receptors are found very early in developing embryos (for examples, in the urogenital tract [28] and in the brain [29] of domestic hen embryos). Although we are not aware of any studies that looked for progesterone receptors and enzymes at the embryonic stage in altricial birds, it has been shown that androgen and oestrogen receptors are abundantly expressed in the brains of altricial species' embryos [18]. We can assume that it is probably the same for progesterone receptors. Progesterone is therefore likely to be a key hormone for avian embryos during early development. Progesterone has been implicated in the regulation of social interactions, parental behaviours and body mass in birds [30]–[33]. However, until now progesterone has received very little attention in the context of ecological and evolutionary studies.

Injection of high levels of exogenous progesterone into yolk caused significant reductions in body and shank mass and length in chick embryos [32]. Plasma progesterone levels and body mass gain have also been observed to be inversely correlated in altricial chicks (Nazca boobies *Sula granti* [33]). Moreover, plasma progesterone is at its highest level when body mass is lowest, and at its lowest level when body mass is highest, in female king penguins *Aptenodytes patagonicus* [30]. Even if the effects of progesterone change with developmental phase and differ between altricial and precocial species, the relationship between progesterone and body mass appears to be consistently negative. Given the positive influence of body mass on the outcome of interactions among nestling birds [34], we hypothesized that females deposit higher progesterone levels in the last egg(s) to hatch compared to the first one(s) when the future parental ability to feed the clutch is likely to be poor. This differential investment could give an advantage to the first chick(s) and accelerate brood reduction. Conversely, females should deposit lower progesterone levels in the last egg(s) to hatch compared to the older sibling(s) when future parental ability is good and brood reduction is not necessary.

We tested this hypothesis in rockhopper penguins *Eudyptes chrysocome chrysocome*, a species exhibiting both reversed hatching asynchrony and brood reduction [35], [36]. In this species, the larger second-laid egg (B-egg) hatches before the smaller first-laid egg (A-egg) although incubation starts only at clutch completion. Moreover, although both eggs commonly hatch, the chick hatched from the A-egg generally dies of starvation within days of hatching. However, in the Falkland Islands, parents can sometimes fledge both chicks of the clutch [37], [38], although this phenomenon has not been quantified in detail and remains poorly understood (but see [38]). In this population, we predicted that the difference in yolk progesterone levels between A- and B-eggs should change with the female's future ability to feed chicks (measured as her body mass at laying and as the date of laying onset).

Since in rockhopper penguins, females are the only parents feeding chicks during the first few weeks of life [39], we did not expect males to have a strong impact on this mechanism. Lightweight females should benefit from early brood reduction via an enhancement of the size hierarchy within the clutch, while heavy females could attempt to fledge two chicks. Heavy females were therefore expected to deposit proportionally more progesterone in the B-egg than in the A-egg while light females should deposit less progesterone in the B-egg than in the A-egg.

For many marine birds including penguins, prey availability varies according to the life cycle of the prey itself and to predators' activity around the colony [40]–[43]. Such factors, which vary with the season, drive the breeding phenology of the species [44] and probably the highly synchronised laying and hatching periods of colonial seabirds such as rockhopper penguins [45], [46]. Since this synchrony is an important component of the breeding success of colonial birds, we also examined whether the date of laying onset (i.e. the day the first egg of the clutch was laid) influenced yolk progesterone levels. We hypothesized that early breeders, which benefit from better food availability during chick feeding, would deposit proportionally more progesterone in the B-egg than in the A-egg compared to late breeders.

## Materials and Methods

### Ethical statement

The study was performed under proper legislation of the Belgian and Flemish law and was approved by the ethical committee on animal experimentation (ECD, ID numbers: 2011/44 and 2011/45). All work was conducted under a research license granted by the Environmental Planning Department of the Falkland Islands Government (Research Licence No: R06/2009). This license covered animal welfare in addition to collection of the egg samples. The methods that we used (nest check, female capture, egg collection) did not cause any desertion from nestling activity or mortality. Collected eggs were replaced with eggs found outside their own nest that we considered as lost by their original parents in order to avoid affecting the breeding success of the colony.

### Study site and birds

The study was carried out in the “Settlement colony” on New Island, Falkland Islands (51°43′S, 61°17′W) from late October to early November 2009. This colony has approximately 5000 pairs of breeding southern rockhopper penguins. Their breeding biology at this colony has been described by Poisbleau et al. [37]. Briefly, males arrive at the colony first (early October) and establish nest sites. Females arrive a few days later, for pairing and copulation. Laying intervals are highly standardized in this species. Within clutches, the B-egg is generally laid four days after the A-egg.

After the arrival of the first males, we visited study sites daily; initially to mark active nests and subsequently to follow the egg laying.

### Egg collection

When a new A-egg was detected in a study nest, we collected it. We replaced this egg with an egg found outside its own nest that we considered as lost by its original parents. Afterwards, we checked the nest daily until the laying of the B-egg. We collected the B-egg as soon as it was detected in the study nest and replaced it with a lost egg. As incubation in rockhopper penguins typically does not start before clutch completion [39], the A-eggs were not incubated and the B-eggs were not incubated for longer than 24 h at collection. We therefore assumed that embryo development and (potential) change in progesterone concentrations (see [27]) had not yet begun and that the eventual difference between A- and B-egg progesterone concentrations was not due to the procedure of egg collection. No embryo development was observed during the preparation of any of the collected eggs.

In total, we collected 60 whole clutches. After collection, we weighed the eggs to the nearest 0.1 g using a digital balance and froze them whole at −20°C.

### Egg preparation

The same method was used to prepare all frozen eggs for subsequent hormone analysis [47], [48]. We first removed the shell while the egg was still frozen. Then, we separated the yolk from the albumen by taking advantage of the fact that albumen thaws more quickly than yolk [23], [49]. We recorded the mass of the yolk to the nearest 0.1 g using a digital balance. Since progesterone concentrations are not homogeneous within the yolk, being the highest in the external layers [23], [50], [51], we carefully homogenized the yolk by swirling it with a mini-spatula. We therefore obtained a yolk sample representative of the whole yolk. A small quantity of each homogenized yolk was transferred to a 1.5-ml Eppendorf tube and stored at -20°C until hormone analysis.

### Hormone analysis

Yolk concentrations of progesterone were determined by radioimmunoassay at the Centre d'Etudes Biologiques de Chizé (CEBC-CNRS) according Mauget et al. [52] adapted for yolk.

Briefly, 200 mg of each sample (weighed to the nearest 0.01 mg) were homogenised in 1 ml of distilled water. Steroids were extracted by adding 3 ml of diethyl-ether to 100 µl of the resulting emulsion, vortexing and centrifuging. The diethyl-ether phase was decanted and poured off after snap freezing the tube in an alcohol bath at −40°C. This was done twice for each sample and the resultant was then evaporated. Extraction efficiency was estimated by adding 1,000 counts per minute (CPM) of ^3^H-progesterone to samples. Recoveries were higher than 90%. The dried extract was re-dissolved in 200 µL of phosphate buffer and incubated overnight at 4°C with 7000 CPM of ^3^H-progesterone (Perkin Elmer, US) and a polyclonal rabbit antiserum against progesterone-11-HS-BSA (Paris, France). Bound and free fractions were separated by adding dextran-coated charcoal. After centrifugation, activity of bound fractions was counted with a Tri-carb 1600TR liquid scintillation counter (Packard). Cross-reactions of progesterone antiserum were as follows: deoxycorticosterone (3%), 6β-hydroprogesterone (1.8%), 5α-dihydroprogesterone (1.6%), 20α-dihydroprogesterone (0.5%), corticosterone (0.3%), pregnanedione (0.2%), Δ5-pregnolone (0.07%), testosterone (0.03%), 17α-hydroprogesterone (0.02%), estradiol (<0.02%), cortisol (<0.01%), aldosterone (<0.01%). Tests were performed to validate the progesterone assay on egg yolk samples. Inter- and intra-assay variations were 12.5% and 10.2% respectively. Four assays were performed. Two yolk samples were serially diluted in the assay buffer and their displacement curves were parallel to the standard curve. Progesterone recovery mean in yolk samples spiked with standard was 104%. The lowest detectable concentration was 0.18 ng/mL of egg yolk.

### Female captures

The 60 females from which we collected the clutch were captured the day they laid their A-eggs (i.e. date of laying onset). To minimize disturbance, the bird's head was covered during the measurements. Bill length and bill depth were measured to the nearest 0.1 mm using callipers following Poisbleau et al. [53]. We weighed each bird to the nearest 20 g with an electronic balance (female body mass at laying). The sex of birds was accessed via a visual comparison within pairs, the bill being larger in males than females [53], and afterwards verified using behaviour. Both males and females were at the nest during captures.

During the laying interval, A-eggs are guarded but not effectively incubated [54], [55]. If the female was standing on the nest when we captured it, the male immediately took its place. All females returned to their normal duties within a few minutes of release. Since the formation of the B-egg yolk ends before the A- egg is laid [56]–[58], it is highly unlikely that female capture influenced hormone levels in B-egg yolks.

### Statistical analysis

Due to the fact that A- and B-eggs vary in size and mass in this species [37], a higher progesterone concentration in A-eggs than in B-eggs does not necessarily mean a higher quantity of progesterone for the former. We therefore calculated the total yolk progesterone per yolk (in ng) by multiplying yolk mass (in g) and yolk progesterone concentration (in pg/mg). The laying date of the first-laid egg of the clutch (A-egg; the date of laying onset) was used for all the statistical analyses.

Statistical tests were performed in SPSS 16.0. Yolk progesterone concentrations and total yolk progesterone followed a normal distribution (Kolmogorov-Smirnov tests, *P*>0.05). We first tested for variation in yolk progesterone concentration and total yolk progesterone using General Linear Models (GLMs) with egg category (A- or B-eggs) as a fixed factor and date of laying onset and female body mass at laying as covariates. Secondly, we calculated intra-clutch ratios (A-egg: B-egg) for yolk progesterone concentration and for total yolk progesterone in each clutch. These ratios followed normal distributions (Kolmogorov-Smirnov tests, *P*>0.05). They were then related to the date of laying onset and female body mass at laying using GLMs. All GLMs were initiated with full-factorial interactions, and subjected to backwards stepwise model simplification. We used Pearson's correlations to explore the correlation in yolk progesterone concentration and total yolk progesterone between A- and B-eggs of the same clutch and also to explore the association between the date of laying onset and female body mass at this date. We used paired *t*-tests to test whether yolk progesterone concentration and total yolk progesterone were significantly different between A- and B-eggs of the same clutch, and univariate linear regressions to explore the relationships of yolk progesterone concentration and total yolk progesterone with date of laying onset and with female body mass. Values are presented as means ± standard deviation (SD).

## Results

Neither yolk progesterone concentrations nor total yolk progesterone were correlated between A- and B-eggs of the same clutch (Pearson correlation: *r*  =  −0.114, *P* = 0.387 and *r*  =  −0.015, *P* = 0.910, respectively). B-eggs had significantly higher yolk progesterone concentrations than A-eggs (mean 265.2±59.2 pg/mg and 227.5±49.6 pg/mg, respectively; table 1; paired t-tests: *t*_59_ = 3.590, *P* = 0.001). This difference remained for total yolk progesterone (5891.7±1359.0 ng and 4528.7±1295.6 ng, respectively; table 1; paired t-tests: *t*_59_ = 5.581, *P*<0.001).

**Table 1:** Test of the variation in yolk progesterone (in pg/mg) concentrations and total yolk progesterone (in ng).

	Yolk progesterone concentration				Total yolk progesterone			
Parameter	df	*F*	*P*	*η_p_^2^*	df	*F*	*P*	*η_p_^2^*
Egg category	1,115	59.652	<0.001	0.342	1,115	61.877	<0.001	0.350
Female body mass	1,115	5.004	0.027	0.042	1,115	5.827	0.017	0.048
Laying onset	1,115	1.469	0.228	0.013	1,115	1.812	0.181	0.016
Egg category * Laying onset	1,115	41.327	<0.001	0.264	1,115	31.666	<0.001	0.216

Results of GLMs with egg category (A- or B-eggs) and as a fixed factor and female body mass and date of laying onset as covariates. *n* = 120 for both dependent variables. Only significant interactions are shown in these models, while other non-significant ones were removed from the model during the backwards-stepwise procedure. As a measure of effect sizes we used partial Eta-Square values (*η_p_^2^*; i.e. the proportion of the effect + error variance that is attributable to the effect) for the factors and covariates tested with a GLM.

**Figure 1:**
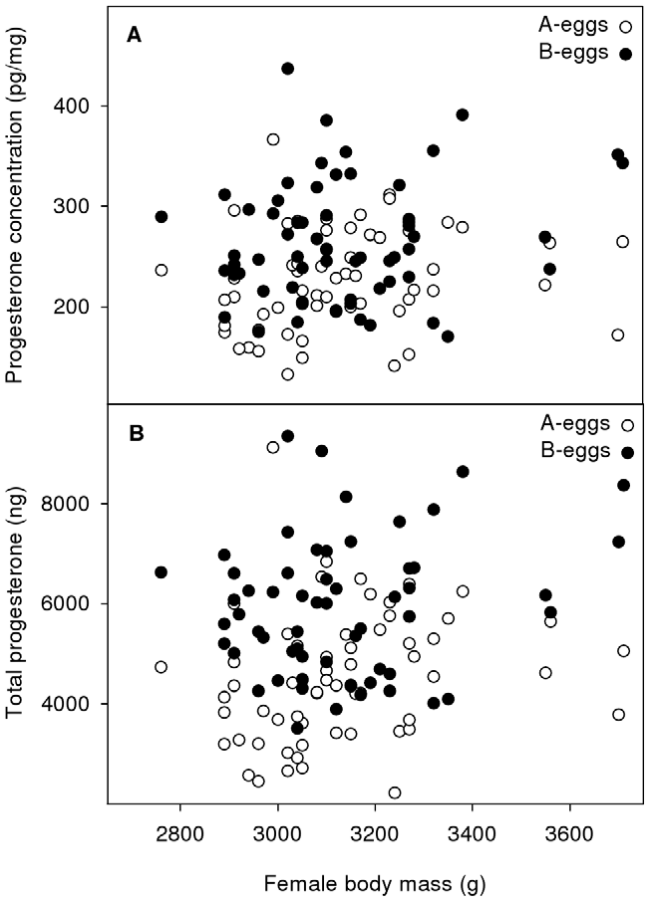
Relationship between female body mass and (a) yolk progesterone concentration and (b) total yolk progesterone.

Female body mass at laying explained a very small part of the variation in both these outcomes (table 1), with both yolk progesterone concentrations and total yolk progesterone slightly increasing with female body mass at laying (univariate linear regression: *t* = 1.807, *P* = 0.073 and *t* = 1.885, *P* = 0.062, respectively; figure 1). However, the intra-clutch ratios of yolk progesterone concentrations and total yolk progesterone between A- and B-eggs were not related to female body mass at laying (*F*_1,57_ = 0.229, *P* = 0.634, *η_p_^2^* = 0.004 for yolk progesterone concentration and *F*_1,57_ = 0.642, *P* = 0.426, *η_p_^2^* = 0.011 for total yolk progesterone when this covariate was removed from the model).

**Figure 2:**
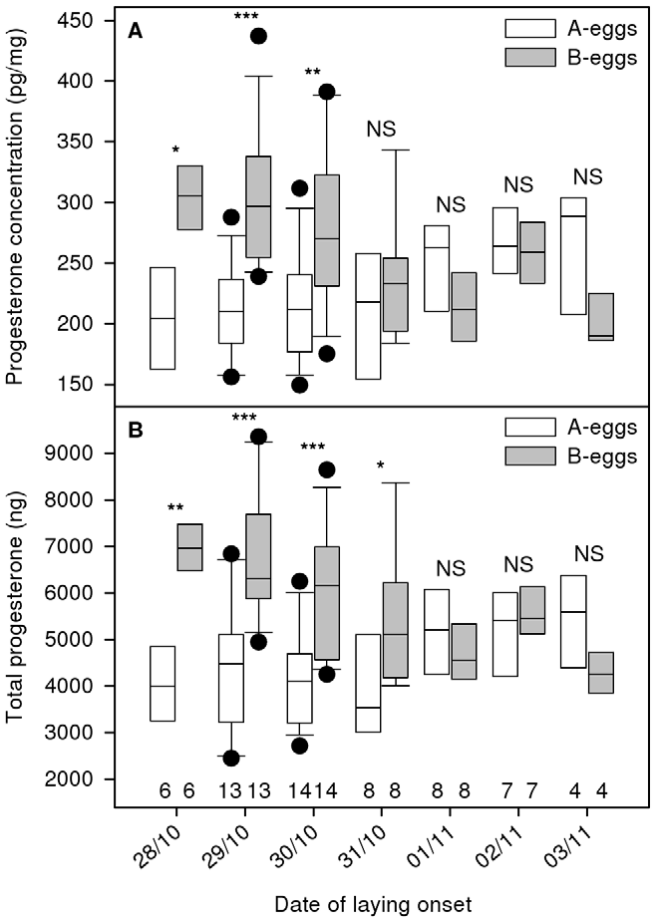
Variation in (a) yolk progesterone concentration and (b) total yolk progesterone according to egg category (white boxes: A-eggs; grey boxes: B-eggs) and the date of laying onset. Boxes show medians, 25% and 75% quartiles for each day; whiskers indicate the range between the 10^th^ and 90^th^ percentiles. •: Data outside the 10^th^ and 90^th^ percentiles. Sample sizes are given under the boxes in the lower frame. Significance of paired t-tests with egg category (A- or B-eggs) as the grouping variable is presented above respective boxes for each date of laying onset; NS: *P*>0.05, *: 0.01<*P*≤0.05, **: 0.001<*P*<0.01, ***: P<0.001.

**Figure 3:**
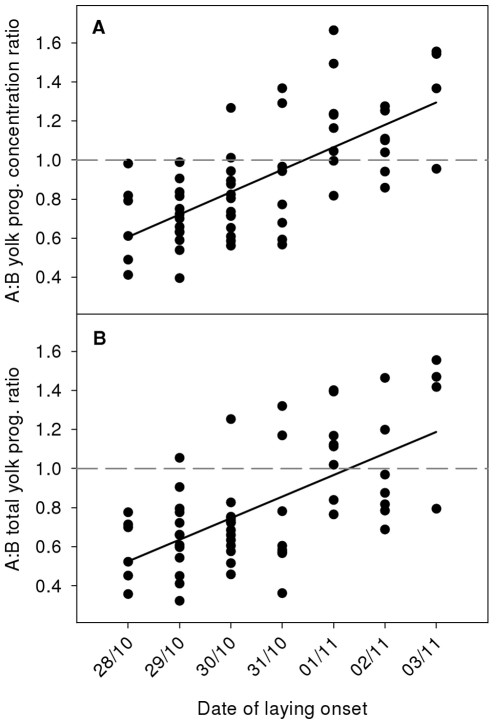
Intra-clutch ratios of (a) yolk progesterone concentration and (b) total yolk progesterone between A- and B-eggs according to the date of laying onset. The solid lines are univariate regression models predicting ratios of yolk progesterone concentration or total yolk progesterone from the date of laying onset and dashed lines represent the ratio expected in the absence of any difference between A- and B-eggs. *n* = 60 clutches.

Both yolk progesterone concentrations and total yolk progesterone changed significantly with the date of laying onset in interaction with egg category (table 1). Yolk progesterone concentrations and total yolk progesterone increased with time for A-eggs (univariate linear regression: *t* = 3.881, *P*<0.001 and *t* = 2.909, *P* = 0.005, respectively; figure 2), while they decreased with time for B-eggs (univariate linear regression: *t*  =  −5.008, *P*<0.001 and *t*  =  −4.955, *P*<0.001, respectively; figure 2). Consequently, the proportion of clutches with lower yolk progesterone concentrations and total yolk progesterone in the B-egg than in the A-egg increased with the date of laying onset. The intra-clutch ratios were significantly related to the date of laying onset (*F*_1,58_ = 48.468, *P*<0.001, *η_p_^2^* = 0.455 for yolk progesterone concentration and *F*_1,58_ = 37.311, *P*<0.001, *η_p_^2^* = 0.391 for total yolk progesterone): early clutches had proportionally more progesterone in the B-egg compared to the A-egg while late clutches had proportionally less progesterone in the B-egg (figure 3).

The date of laying onset was not related to female body mass at laying (Pearson correlation: r = 0.018, P = 0.894).

## Discussion

We tested whether yolk progesterone levels differed between eggs according to the future parental ability to feed the entire clutch in wild rockhopper penguins. Overall, we observed that B-eggs had significantly higher yolk progesterone levels (both in concentrations and total amounts) than A-eggs in rockhopper penguins. However, this general result was reversed in many clutches. This variation in the intra-clutch pattern of yolk hormone levels seems less common for the other hormones measured in this species [48], [59], [60]. Among 20 clutches collected in 2007, only one had slightly higher testosterone and dihydrotestosterone (DHT) levels in the A-egg than in the B-egg and none had higher androstenedione (A4) levels [59]. Whatever the positive or negative effects of measured yolk hormone (other than progesterone) levels on embryos and chicks, the constancy of their intra-clutch ratios showed that one chick category was always favoured over the other one. This is clearly not the case for progesterone, yet it is a precursor of testosterone. Since a recent study showed that the corticosterone measurements reported on rockhopper penguin yolks [48] in reality also reflect high concentrations of yolk progesterone and its precursors [61], it is not relevant to further compare our present findings with corticosterone levels measured on the same species [48].

We proposed that females could adjust the levels of yolk progesterone (as of other yolk hormones, see review in [19]) over the laying sequence to reduce or enhance the effects of the size hierarchy between siblings according to the breeding conditions they experience during egg formation and/or they expect to experience during chick rearing. Breeding conditions here were assessed through both female body mass at laying and date of laying onset.

We detected a slight general increase of yolk progesterone levels with female body mass at laying. However, the intra-clutch ratios of yolk progesterone levels did not vary with female body mass, implying that the increase in yolk progesterone levels with female body mass increase was similar for A- and B-eggs. Female body mass is therefore not likely to affect the size hierarchy through progesterone levels. These findings do not follow those obtained on testosterone and A4 levels for the same species [60]: androgen levels were significantly negatively related to female body mass in B-eggs while these measurements varied independently for A-eggs [60]. These results suggest that, if female body mass acts as a driver of brood reduction in rockhopper penguins, maternal yolk progesterone is not mediating this relationship, while maternal yolk androgen deposition could be. Given that yolk androgens positively influence the outcome of sibling competition through potential effects on embryonic developmental time and on chick begging behaviour, food competitiveness and growth rates (see review in [17]), this pattern of yolk androgen deposition between eggs favours B-eggs and probably ensures a quicker brood reduction in females with low body mass.

Yolk progesterone levels increased with the date of laying onset for A-eggs while they decreased for B-eggs. We therefore observed that the within-clutch difference in yolk progesterone levels between A- and B-eggs gradually changed with time. At the beginning of the laying period, all clutches had higher yolk progesterone in the B-egg than in the A-egg, but the trend was reversed for clutches laid after the middle of the laying period, with some clutches having more yolk progesterone in the A-egg than in the B-egg. For androgens, it was observed that late clutches had proportionally higher levels in the B-egg compared to the A-egg than early clutches [59]. Interestingly, we note that yolk androgen deposition is modulated only in B-eggs, while yolk progesterone is modulated simultaneously in both egg categories.

Hatching is highly standardized in rockhopper penguins, with the A-egg generally hatching one day later than the B-egg, but occasionally hatching on the same day (22%) or two days later (18%, *n* = 92 clutches, MP unpublished data and [37]). Since yolk progesterone levels have been observed to be negatively linked to nestling growth [32], lower yolk progesterone levels in A-eggs (the smallest and the second to hatch) than in B-eggs (the biggest and the first to hatch) for early clutches may be a way of compensating for the initial size hierarchy between A- and B-eggs. Because Nazca booby chicks with a sibling have lower plasma progesterone levels and higher mass increase than unchallenged chicks, Tarlow et al. [33] suggested that plasma progesterone could be regulated to promote exaggerated mass gain in socially challenged chicks. We therefore propose here that maternal yolk progesterone could also be regulated to influence mass gain of the embryos and chicks according to the parents' future ability to feed the entire clutch.

The fact that the pattern of deposition according to the date of laying onset is opposite between the two eggs of the clutch (increasing with time for A-eggs but decreasing for B-eggs) suggests that the mechanism governing deposition of yolk progesterone differs between egg categories. More specifically, maternal yolk progesterone deposition shows that the growth of embryos from A-eggs may be favoured at the beginning of the laying period, compared to embryos from B-eggs while neither egg category may be favoured in regard of yolk progesterone deposition at the end of the laying period (see figure 2). Since it does not seem adaptive to decrease the growth rates of either chick, even if it may enhance survival of the other, we suggest that yolk progesterone levels could be decreased in A-eggs that are laid early in the breeding season to give those chicks a better survival chance. The high yolk progesterone levels in their B-egg siblings might therefore only be a compensatory consequence because the female has to evacuate a certain amount of progesterone. However, the fact that yolk progesterone is produced in follicles, different for each yolk, [25] and that its levels do not mirror female plasma levels (see [23]) do not support this hypothesis. Alternatively, the low yolk progesterone levels in A-eggs, compared to their B-egg siblings, might be an additional adaptive mechanism to increase the size hierarchy between siblings if females would be able to actively control hormone deposition (see discussion on the subject in [18]).

In conclusion, a differential investment with lower yolk progesterone levels in the second eggs to hatch (A-eggs) should be associated with a reduction in the advantage enjoyed by the first chick and with a reduced frequency of brood reduction when breeding conditions are expected to be good during chick rearing. Conversely, lower (or similar) progesterone levels in the first eggs to hatch (B-eggs) should allow the negative effects of hatching asynchrony to be expressed. Brood reduction would be accelerated in favour of B-eggs, the eggs with the highest expected value, when breeding conditions are expected to be bad during chick rearing. Therefore, the reported within-clutch difference in yolk progesterone supports the hypothesis that females can strategically deposit different progesterone levels within the clutch according to the future parental ability to feed the clutch. The present study stresses the importance of further investigation of role of progesterone in both altricial and precocial species and of exploring how the different hormones present in the yolk interact to affect brood reduction, chick development and behaviour in general.

## References

[1] Rhymer JM (1992). An experimental study of geographic variation in avian growth and development.. Journal of Evolutionary Biology.

[2] Sedinger JS, Flint PL, Lindberg MS (1995). Environmental influence on life-history traits: growth, survival, and fecundity in black brant (*Branta bernicla*).. Ecology.

[3] Reed ET, Gauthier G, Pradel R, Lebreton J-D (2003). Age and environmental conditions affect recruitment in greater snow geese.. Ecology.

[4] Lindström J (1999). Early development and fitness in birds and mammals.. Trends in Ecology and Evolution.

[5] Shine R, Downes SJ (1999). Can pregnant lizards adjust their offspring phenotypes to environmental conditions?. Oecologia.

[6] Metcalfe NB, Monaghan P (2001). Compensation for a bad start: grow now, pay later?. Trends in Ecology and Evolution.

[7] Royle NJ, Lindström J, Metcalfe NB (2005). A poor start in life negatively affects dominance status in adulthood independent of body size in green swordtails *Xiphophorus helleri*.. Proceeding of the Royal Society of London series B - Biological Sciences.

[8] Mousseau TA, Fox CW (1998). Maternal effects as adaptations..

[9] Mousseau TA, Fox CW (1998). The adaptive significance of maternal effects.. Trends in Ecology and Evolution.

[10] Zach R (1982). Hatching asynchrony, egg size, growth, and fledging in tree swallows.. The Auk.

[11] Slagsvold T, Sandvik J, Rofstad G, Lorentsen Ö, Husby M (1984). On the adaptive value of intraclutch egg-size variation in birds.. The Auk.

[12] Stokland JN, Amundsen T (1988). Initial size hierarchy in broods of the shag: relative significance of egg size and hatching asynchrony.. The Auk.

[13] Lack D (1947). The significance of clutch size.. Ibis.

[14] O'Connor RJ (1978). Brood reduction in birds: selection for fratricide, infanticide and suicide?. Animal Behaviour.

[15] Clark AB, Wilson DS (1981). Avian breeding adaptations: hatching asynchrony, brood reduction, and nest failure.. The Quarterly Review of Biology.

[16] Groothuis TGG, von Engelhardt N (2005). Investigating maternal hormones in avian eggs: measurement, manipulation, and interpretation.. Annals of the New York Academy of Sciences.

[17] Gil D (2008). Hormones in avian eggs: physiology, ecology and behavior.. Advances in the Study of Behavior.

[18] Groothuis TGG, Schwabl H (2008). Hormone-mediated maternal effects in birds: mechanisms matter but what do we know of them?. Philosophical Transactions of the Royal Society of London, serie B: Biological Sciences.

[19] Groothuis TGG, Müller W, von Engelhardt N, Carere C, Eising C (2005). Maternal hormones as a tool to adjust offspring phenotype in avian species.. Neuroscience and Biobehavioral Reviews.

[20] Love OP, Gilchrist HG, Bêty J, Wynne-Edwards KE, Berzins L (2009). Using life-histories to predict and interpret variability in yolk hormones.. General and Comparative Endocrinology.

[21] Schwabl H, Mock DW, Gieg JA (1997). A hormonal mechanism for parental favouritism.. Nature.

[22] Andersson S, Uller T, Lõhmus M, Sundström F (2004). Effects of egg yolk testosterone on growth and immunity in a precocial bird.. Journal of Evolutionary Biology.

[23] Lipar JL, Ketterson ED, Nolan V, Casto JM (1999). Egg yolk layers vary in the concentration of steroid hormones in two avian species.. General and Comparative Endocrinology.

[24] Bahr JM, Wang S-C, Huang MY, Calvo FO (1983). Steroid concentrations in isolated theca and granulosa layers of preovulatory follicles during the ovulatory cycle of the domestic hen.. Biology of Reproduction.

[25] Huang ESR, Kao KJ, Aarvak T, Nalbandov AV (1979). Synthesis of sex steroids by cellular-components of chicken follicles.. Biology of Reproduction.

[26] Lipar JL (2001). Yolk steroids and the development of the hatching muscle in nestling European starlings.. Journal of Avian Biology.

[27] Paitz RT, Bowden RM, Casto JM (2011). Embryonic modulation of maternal steroids in European starlings (*Sturnus vulgaris*).. Proceeding of the Royal Society of London series B - Biological Sciences.

[28] Gasc J-M (1991). Distribution and regulation of progesterone receptor in the urogenital tract of the chick embryo.. Anatomy and Embryology.

[29] Camacho-Arroyo I, González-Arenas A, González-Agüero G, Guerra-Araiza C, González-Morán G (2003). Changes in the content of progesterone receptor isoforms and estrogen receptor alpha in the chick brain during embryonic development.. Comparative Biochemistry and Physiology Part A.

[30] Cherel Y, Mauget R, Lacroix A, Gilles J (1994). Seasonal and fasting-related changes in circulating gonadal steroids and prolactin in king penguins, *Aptenodytes patagonicus*.. Physiological Zoology.

[31] Blas J, López L, Tanferna A, Sergio F, Hiraldo F (2010). Reproductive endocrinology of wild, long-lived raptors.. General and Comparative Endocrinology.

[32] Renden JA, Benoff FH (1980). Effects of progesterone on developing chick embryos.. Poultry Science.

[33] Tarlow EM, Wikelski M, Anderson DJ (2001). Hormonal correlates of siblicide in Galápagos nazca boobies.. Hormones and Behavior.

[34] Mock DW, Parker GA (1997). The evolution of sibling rivalry: Oxford University Press.

[35] Gwynn AM (1953). The egg-laying and incubation periods of rockhopper, macaroni and gentoo penguins.. A N A R E Reports Series B.

[36] Lamey TC, Davis LS, Darby JT (1990). Hatch asynchrony and brood reduction in penguins.. Penguin biology.

[37] Poisbleau M, Demongin L, Strange IJ, Otley H, Quillfeldt P (2008). Aspects of the breeding biology of the southern rockhopper penguin *Eudyptes c. chrysocome* and new consideration on the intrinsic capacity of the A-egg.. Polar Biology.

[38] Demongin L, Poisbleau M, Raya Rey A, Schiavini A, Quillfeldt P (2010). Geographical variation in egg size dimorphism in rockhopper penguins.. Polar Biology.

[39] Williams TD (1995). The penguins..

[40] Ashmole NP (1963). The regulation of numbers of tropical oceanic birds.. Ibis.

[41] Birt VL, Birt TP, Goulet D, Cairns DK, Montevecchi WA (1987). Ashmole's halo: direct evidence for prey depletion by a seabird.. Marine Ecology Progress Series.

[42] Charrassin J-B, Park Y-H, Le Maho Y, Bost C-A (2002). Penguins as oceanographers unravel hidden mechanisms of marine productivity.. Ecology Letters.

[43] Ainley DG, Ribic CA, Ballard G, Heath S, Gaffney I (2004). Geographic structure of Adélie penguin populations: overlap in colony-specific foraging areas.. Ecological Monographs.

[44] Durant JM, Hjermann DØ, Anker-Nilssen T, Beaugrand G, Mysterud A (2005). Timing and abundance as key mechanisms affecting trophic interactions in variable environments.. Ecology Letters.

[45] Gochfeld M, Burger J, Olla BL, Winn HE (1980). Mechanisms and adaptive value of reproductive synchrony in colonial seabirds.. Behavior of marine animals: current perspectives in research.

[46] Wittenberger JF, Hunt GL, Farner DS, King JR, Parkes KC (1985). The adaptive significance of coloniality in birds.. Avian biology VIII: Academic Press.

[47] Poisbleau M, Demongin L, Angelier F, Dano S, Lacroix A (2009). What ecological factors can affect albumen corticosterone levels in the clutches of seabirds? Timing of breeding, disturbance and laying order in rockhopper penguins (*Eudyptes chrysocome chrysocome*).. General and Comparative Endocrinology.

[48] Poisbleau M, Demongin L, Trouve C, Quillfeldt P (2009). Maternal deposition of yolk corticosterone in clutches of southern rockhopper penguins (*Eudyptes chrysocome chrysocome*).. Hormones and Behavior.

[49] Lipar JL, Ketterson ED, Nolan V (1999). Intraclutch variation in testosterone content of red-winged blackbird eggs.. The Auk.

[50] Möstl E, Spendier H, Kotrschal K (2001). Concentration of immunoreactive progesterone and androgens in the yolk of hens' eggs (*Gallus domesticus*).. Wiener Tierarztliche Monatsschrift.

[51] Hackl R, Bromundt V, Daisley J, Kotrschal K, Möstl E (2003). Distribution and origin of steroid hormones in the yolk of Japanese quail eggs (*Coturnix coturnix japonica*).. Journal of Comparative Physiology B.

[52] Mauget R, Jouventin P, Lacroix A, Ishii S (1994). Plasma LH and steroid hormones in king penguin (*Aptenodytes patagonicus*) during the onset of the breeding cycle.. General and Comparative Endocrinology.

[53] Poisbleau M, Demongin L, van Noordwijk HJ, Strange IJ, Quillfeldt P (2010). Sexual dimorphism and use of morphological measurements to sex adults, immatures and chicks of rockhopper penguins.. Ardea.

[54] Warham J, Stonehouse B (1975). The crested penguins.. The biology of penguins.

[55] Williams TD (1989). Aggression, incubation behaviour and egg-loss in macaroni penguins, *Eudyptes chrysolophus*, at South Georgia.. Oikos.

[56] Astheimer LB, Grau CR (1985). The timing and energetic consequences of egg formation in the Adélie penguin.. The Condor.

[57] Crossin GT, Trathan PN, Phillips RA, Dawson A, Le Bouard F (2010). A carryover effect of migration underlies individual variation in reproductive readiness and extreme egg size dimorphism in macaroni penguins.. The American Naturalist.

[58] Grau CR (1982). Egg formation in Fiordland crested penguins (*Eudyptes pachyrhynchus*).. The Condor.

[59] Poisbleau M, Demongin L, Chastel O, Eens M, Quillfeldt P (2011). Yolk androgen deposition in rockhopper penguins, a species with reversed hatching asynchrony.. General and Comparative Endocrinology.

[60] Poisbleau M, Carslake D, Demongin L, Eens M, Chastel O (2011). Yolk androgen deposition without an energetic cost for female rockhopper penguins: a compensatory strategy to accelerate brood reduction?. Biology Letters.

[61] Quillfeldt P, Poisbleau M, Parenteau C, Trouvé C, Demongin L (2011). Measuring corticosterone in seabird egg yolk and the presence of high yolk gestagen concentrations.. General and Comparative Endocrinology.

